# Insights into alloy/oxide or hydroxide interfaces in Ni–Mo-based electrocatalysts for hydrogen evolution under alkaline conditions

**DOI:** 10.1039/d2sc06298d

**Published:** 2023-01-19

**Authors:** Min Luo, Jietian Yang, Xingang Li, Miharu Eguchi, Yusuke Yamauchi, Zhong-Li Wang

**Affiliations:** a Tianjin Key Laboratory of Applied Catalysis Science and Technology, School of Chemical Engineering and Technology, Tianjin University Tianjin 300072 China wang.zhongli@tju.edu.cn; b JST-ERATO Yamauchi Materials Space-Tectonics Project, International Center for Materials Nanoarchitectonics (WPI-MANA), National Institute for Materials Science (NIMS) 1-1Namiki Tsukuba Ibaraki 305-0044 Japan; c School of Chemical Engineering and Australian Institute for Bioengineering and Nanotechnology (AIBN), The University of Queensland Brisbane QLD 4072 Australia

## Abstract

Nickel–molybdenum (Ni–Mo) alloys are promising non-noble metal electrocatalysts for the hydrogen evolution reaction (HER) in alkaline water; however, the kinetic origins of their catalytic activities still remain under debate. In this perspective, we systematically summarize the structural characteristics of Ni–Mo-based electrocatalysts recently reported and find that highly active catalysts generally have alloy–oxide or alloy–hydroxide interface structures. Based on the two-step reaction mechanism under alkaline conditions, water dissociation to form adsorbed hydrogen and combination of adsorbed hydrogen into molecular hydrogen, we discuss in detail the relationship between the two types of interface structures obtained by different synthesis methods and their HER performance in Ni–Mo based catalysts. For the alloy–oxide interfaces, the Ni_4_Mo/MoO_*x*_ composites produced by electrodeposition or hydrothermal combined with thermal reduction exhibit activities close to that of platinum. For only the alloy or oxide, their activities are significantly lower than that of composite structures, indicating the synergistic catalytic effect of binary components. For the alloy–hydroxide interfaces, the activity of the Ni_*x*_Mo_*y*_ alloy with different Ni/Mo ratios is greatly improved by constructing heterostructures with hydroxides such as Ni(OH)_2_ or Co(OH)_2_. In particular, pure alloys obtained by metallurgy must be activated to produce a layer of mixed Ni(OH)_2_ and MoO_*x*_ on the surface to achieve high activity. Therefore, the activity of Ni–Mo catalysts probably originates from the interfaces of alloy–oxide or alloy–hydroxide, in which the oxide or hydroxide promotes water dissociation and the alloy accelerates hydrogen combination. These new understandings will provide valuable guidance for the further exploration of advanced HER electrocatalysts.

## Abstract 2

The activity of Ni–Mo catalysts for the hydrogen evolution reaction originates from the interfaces of alloy–oxide or alloy–hydroxide, in which the oxide or hydroxide promotes water dissociation and the alloy accelerates hydrogen combination.

## Graphical abstract

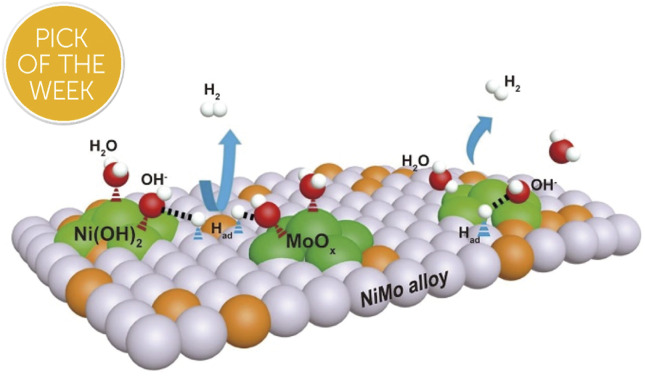

## 1. Introduction

The increasing energy crisis induced by fast depletion of limited fossil fuels and environmental impacts have resulted in an urgent demand for clean and renewable energy resources. Owing to its high energy density and environmentally friendly characteristics, molecular hydrogen is an attractive energy carrier to meet future global energy demands.^1–4^ Electrochemical water splitting that converts water into hydrogen and oxygen is a promising way for sustainable production of hydrogen, especially when it is driven by green electricity from sunlight, wind, hydropower, *etc.* Alkaline water electrolysis is one of the most mature and widely used electrolysis technologies for hydrogen production due to its low-cost components and high durability. However, the sluggish reaction kinetics of the hydrogen evolution reaction (HER) in alkaline media lead to high overpotentials for practical water splitting. To promote the HER kinetics, efficient electrocatalysts are necessary to decrease the reaction overpotentials, thus making the water splitting more energy-saving. As a benchmark HER electrocatalyst, precious metal platinum (Pt)-based materials are almost still the catalysts with the highest intrinsic activity.^5^ Unfortunately, the scarcity and high cost of Pt seriously impede its large-scale applications in the electrocatalytic HER.

To develop efficient and earth-abundant alternatives to Pt as HER electrocatalysts, great efforts have been made to explore transition metal-based electrocatalysts over the past decade, including metal oxides,^6–8^ metal alloys,^9–12^ traditional metal phosphides,^13–17^ carbides,^18,19^ and sulfides.^20–22^ Among them, nickel-based materials are the most used electrocatalysts in alkaline electrolyzers, and especially, nickel–molybdenum (Ni–Mo) bimetallic catalysts exhibit the best HER catalytic performance among all the non-noble metal-based materials.^23–42^ Typically, Ni_4_Mo alloy nanoparticles supported by MoO_2_ cuboids on nickel foam exhibited zero onset overpotential, an overpotential of 15 mV at 10 mA cm^−2^ and a low Tafel slope of 30 mV dec^−1^ in 1 M KOH.^23^ Such performance is comparable to that of Pt and superior to those of state-of-the-art Pt-free electrocatalysts. However, the detailed mechanism for the enhanced activity of Ni–Mo catalysts remains controversial, and several mechanisms have been proposed to account for the high HER activity of Ni–Mo-based electrocatalysts. One prevailing explanation is related to the electronic synergy of Ni and Mo in the alloy, leading to an appropriate hydrogen adsorption energy of the catalyst surface.^43,44^ The second proposed mechanism is that Ni activates water dissociation to produce adsorbed hydrogen species which then transfer to the Mo surface *via* hydrogen spillover to produce molecular hydrogen, called the hydrogen-spillover effect.^45^ And the third explanation is that the intrinsic activity of the Ni–Mo alloy originates from Ni and surface area enhancement through Mo dissolution improves the current density.^46^ These three mechanisms focus on the role of metallic Ni as the primary active site. However, the recent results demonstrated that a Mo oxo species (most likely Mo^3+^) supported on metallic Ni or the Ni_*x*_Mo_*y*_ alloy was responsible for the high activity of a Ni–Mo catalyst for water reduction to form hydrogen.^47^ It can be clearly seen that these mechanisms are inconsistent, indicating the complexity of the Ni–Mo catalytic system. Therefore, it is necessary to further explore the factors that affect the activity of the Ni–Mo catalyst and deeply understand the nature of high activity.

**Fig. 1:**
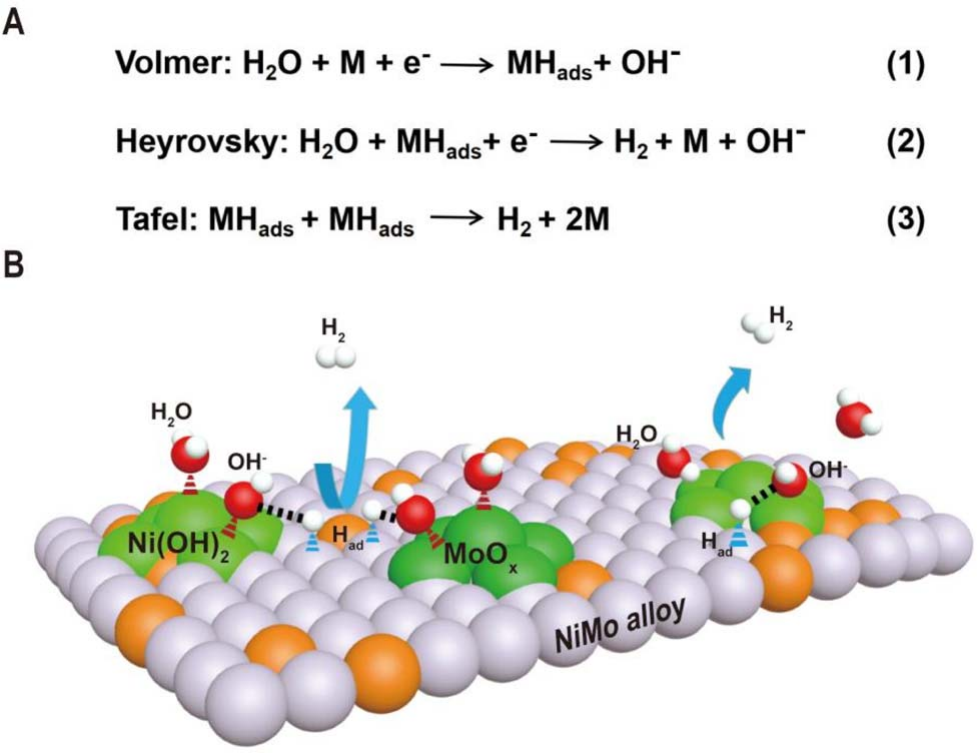
(A) The reaction mechanism for the HER in alkaline electrolytes and two pathways are presented in the forms of the Volmer–Heyrovsky mechanism and the Volmer–Tafel mechanism. (B) Schematic diagram of synergetic catalysis of alloy–oxide or alloy–hydroxide interface structures, in which the oxide or hydroxide promotes water dissociation and the alloy accelerates hydrogen combination.

As shown in Fig. 1, the HER kinetics in alkaline solutions involves two steps: water dissociation to form adsorbed hydrogen (Volmer step) and combination of adsorbed hydrogen into molecular hydrogen (Heyrovsky or Tafel step).^48,49^ Compared to the HER under acidic conditions, where protons are directly used as reactants, the reactants in an alkaline solution switch from protons to water as the hydrogen source, and an additional water dissociation step needs to occur to release protons, which significantly decreases the reaction rate of the HER. As a result, even for Pt-based catalysts, the catalytic performance and kinetics are hindered by the slow rate of water cleavage in the Volmer step. Due to this reason, the reaction rate on Pt is usually 2–3 orders lower in an alkali than that in an acid.^50^ Therefore, efficient HER under alkaline conditions requires that the active sites of the catalyst simultaneously accelerate the water dissociation and hydrogen combination reactions. Interestingly, the decoration of Ni(OH)_2_ on the Pt surfaces has been demonstrated to drastically increase the HER performance of Pt catalysts under alkaline conditions, and it is proposed that Ni(OH)_2_ promotes the cleavage of H–OH bonds in the water dissociation step, while Pt facilitates adsorption and combination of the generated hydrogen intermediates to form H_2_ molecules.^5^ Pt/Ni(OH)_2_-based systems have not only advanced electrocatalytic performances but also provided insight into reaction mechanisms.

Inspired by the fundamental HER mechanism that occurs on Pt/Ni(OH)_2_ interface, we systematically summarize the structural characteristics of Ni–Mo-based electrocatalysts recently reported, and find surprisingly that highly active catalysts generally have alloy–oxide or alloy–hydroxide interface structures. It is not difficult to speculate that the two components at the interface may jointly promote the two-step reaction of the HER in Ni–Mo systems. Based on this understanding, we discuss in detail the relationship between the two types of interface structures obtained by different synthesis methods and their HER performance in Ni–Mo-based catalysts. For the alloy–oxide interfaces, the Ni_4_Mo/MoO_*x*_ (mixed oxide with the Mo ion valence from +4 to +6) composites produced by electrodeposition or hydrothermal combined with thermal reduction exhibit activities close to that of platinum. For only the alloy or oxide, their activities are significantly lower than that of composite structures, indicating the synergistic catalytic effect of binary components. For the alloy–hydroxide interfaces, the activity of the Ni_*x*_Mo_*y*_ alloy with different Ni/Mo ratios is greatly improved by constructing heterostructures with hydroxides such as Ni(OH)_2_ or Co(OH)_2_. In particular, pure alloys obtained by metallurgy must be activated to produce a layer of mixed Ni(OH)_2_ and MoO_*x*_ on the surface to achieve high activity. Therefore, it can be seen that the activity of Ni–Mo catalysts probably originates from the interfaces of alloy–oxide or alloy–hydroxide, in which the oxide or hydroxide promotes water dissociation and the alloy accelerates hydrogen combination. These new understandings will provide valuable guidance for the further exploration of advanced HER electrocatalysts.

## 2. Ni–Mo alloy/oxide interfaces

The preparation of the Ni–Mo alloy can be divided into two categories according to different raw materials: chemical method and metallurgical method. The chemical method generates alloys from Ni and Mo metal salts through a series of chemical reactions, while the metallurgical method directly produces alloys from two metals at high temperatures. Chemical methods are widely used in the synthesis of Ni–Mo alloy catalysts due to their advantages such as simple operation and controllable structure. According to the reaction process, chemical methods can also be divided into two types: one is the direct reduction of metal salts to produce alloys in one step, such as electrochemical reduction deposition, and the other is the two-step method, *i.e.*, the metal salts first form oxide intermediates, such as NiMoO_4_ through a hydrothermal reaction, and then the intermediates are reduced in a hydrogen atmosphere to produce alloys. Since Mo^6+^ is more difficult to reduce than Ni^2+^, only part of Mo ions is reduced to form alloys during the reduction of metal salts or oxide intermediates, and excessive Mo ions will form oxides, resulting in a large number of alloy/oxide interface structures. Therefore, the proportion of Ni/Mo and the reduction conditions determine the composition of the products, mainly including the type of Ni–Mo alloy, the content, and valence of MoO_*x*_. Under general chemical reduction conditions, such as electrochemical reduction or hydrogen reduction below 600 °C, the Ni–Mo alloy is mainly Ni_4_Mo, and MoO_*x*_ is a mixed oxide with the Mo ion valence from +4 to +6. In the following, we will introduce several Ni–Mo-based catalysts prepared by a chemical method, focusing on the interface structures of N_4_Mo/MoO_*x*_ and their HER performances.

**Fig. 2:**
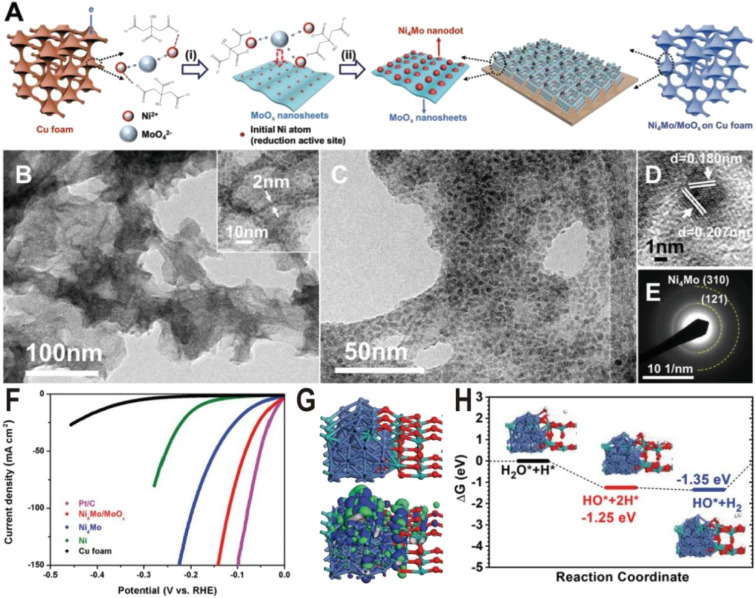
(A) Schematic of the Ni_4_Mo/MoO_*x*_ synthesis route on Cu foam. (i) Induced codeposition; (ii) formation of nanointerfaces with the dispersion of the alloy nanoparticles. (B and C) TEM images, (D) HRTEM image, and (E) SAED pattern of Ni_4_Mo nanoparticles on amorphous MoO_*x*_ nanosheets. (F) Polarization curves of Pt/C, bare Cu foam, Ni, Ni_4_Mo alloy, and Ni_4_Mo/MoO_*x*_. (G) Electronic properties of interface model systems. Brown balls = Cu; cyan balls = Mo; blue balls = Ni; and red balls = O. (H) Free energy pathway, chemisorption, and formation energy for the HER. Reproduced with permission.^51^ Copyright 2019, Wiley-VCH.

The Ni_4_Mo nanodot/amorphous MoO_*x*_ nanosheet interface structure was synthesized on copper foam *via* a one-step electrodeposition process as shown in Fig. 2A.^51^ In the electrodeposition process, the MoO_4_^2−^ anions, Ni^2+^ cations, and the citrate anions first formed [(NiCit)(MoO_*x*_)]_ads_^−^, followed by the reduction of Ni^2+^ to metallic Ni^0^, and then the resulting Ni atoms acted as the active sites for the depositing the Ni_4_Mo alloy on the MoO_*x*_ surface. By changing the ratio of Ni/Mo and electroreduction current density, the samples of Ni_4_Mo and pure Ni were also synthesized for the comparison experiment. The as-synthesized composite catalyst exhibits ultrathin nanosheet morphology (Fig. 2B) and many nanoparticles with a diameter of about 5 nm are homogeneously distributed on the nanosheets (Fig. 2C). The high-resolution transmission electron microscope (HRTEM) image (Fig. 2D) and selective area electron diffraction (SAED) pattern (Fig. 2E) show lattice fringes of (121) and (310) planes of the Ni_4_Mo nanoparticle, while no crystal lattice of MoO_*x*_ nanosheets could be observed, suggesting the amorphous nature of the MoO_*x*_ nanosheets. The strong Raman peaks in the range of 550–1000 cm^−1^ also verify the presence of amorphous molybdenum oxide, and the X-ray photoelectron spectroscopy (XPS) with Ar etching shows that all Ni^2+^ is reduced to Ni^0^, while the Mo element exists not only in the form of Mo^0^ but also oxidized forms including Mo^4+^ and Mo^5+^. Fig. 2F shows the polarization curves of Ni metal, Ni_4_Mo alloy, and Ni_4_Mo/MoO_*x*_ for HER electrocatalysis. The overpotential of Ni_4_Mo/MoO_*x*_ at a current density of 10 mA cm^−2^ is only 16 mV, close to that of Pt/C, and much lower than that of Ni metal (169 mV) and the Ni_4_Mo alloy (40 mV). Evidently, the HER activity of Ni increases substantially by forming an alloy with Mo and is further boosted by constructing the Ni_4_Mo/MoO_*x*_ nanointerfaces, indicating the beneficial bimetallic ligand effect and synergetic effect between the metal and metal oxide. The theoretical study shows that the Ni_4_Mo/MoO_*x*_ has a strong bonding at the interface and the electronic distribution indicates that the Ni_4_Mo alloy is more electron-rich (Fig. 2G), compared with the hole-rich MoO_*x*_. The comparison of projected density of states (PDOS) indicates that the d-band center of Ni at the interface downshifts toward lower energy levels, which optimizes the bonding strength of *H on the alloy. In MoO_*x*_, the deep localized O-2p band overlaps with the Mo-4d-t2g state propelling more electrons towards the Mo-4d-eg level, which leads Mo sites to high binding activities for the O-related species such as *OH intermediates. Therefore, Ni_4_Mo/MoO_*x*_ interface provides a high active area for bond-cleavage of water-splitting under alkaline conditions. The free energy profile for the HER pathway under alkaline conditions is also studied (Fig. 2H), and the Ni_4_Mo/MoO_*x*_ interface shows substantial energetic favorable with reaction energy (−1.35 eV) gained for the HER.

**Fig. 3:**
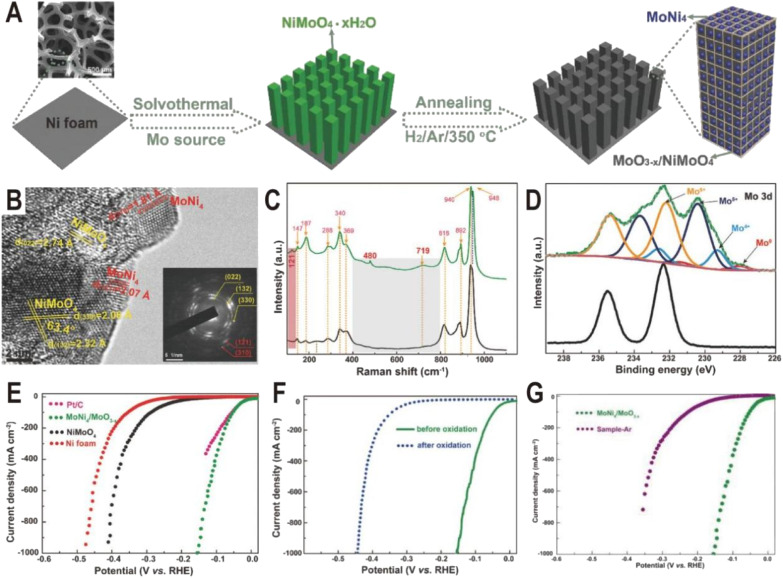
(A) Schematic illustration of the formation of MoNi_4_/MoO_3−*x*_ nanorod arrays on Ni foam. (B) HRTEM image for MoNi_4_/MoO_3−*x*_ (inset: SAED pattern). (C) Raman spectra of NiMoO_4_ (black) and MoNi_4_/MoO_3−*x*_ (green). (D) Mo 3d XPS spectra of NiMoO_4_ (black) and MoNi_4_/MoO_3−*x*_ (green). (E) Polarization curves for Pt/C, Ni foam, NiMoO_4_, and MoNi_4_/MoO_3−*x*_. (F) Polarization curves of MoNi_4_/MoO_3−*x*_ for the HER before and after electrochemical oxidation. (G) Polarization curves of MoNi_4_/MoO_3−*x*_ and NiMoO_4_ annealed in an Ar atmosphere (sample-Ar). Scan rate: 2 mV s^−1^. Reproduced with permission.^52^ Copyright 2017, Wiley-VCH.

In addition to one-step direct reduction, the two-step method is also widely used in the synthesis of the Ni_4_Mo/MoO_*x*_ interface. Typically, NiMoO_4_ nanorod arrays were first grown on Ni foam *via* a solvothermal process using Ni foam itself as a Ni source and ammonium molybdate as a Mo source, and then NiMoO_4_ was partially decomposed to MoNi_4_ nanocrystals and amorphous MoO_3−*x*_ by annealing in a H_2_/Ar atmosphere at 350 °C (Fig. 3A).^52^ The HRTEM image (Fig. 3B) displays clear lattice fringes with distances of 1.81 and 2.07 Å corresponding to the (310) and (121) planes of the MoNi_4_ alloy, suggesting the production of MoNi_4_ nanocrystals. Due to the amorphous phase of MoO_3−*x*_, no continuous lattice fringes are observed in the HRTEM image, but Raman and XPS spectra clearly evidence the existence of MoO_3−*x*_. As shown in Fig. 3C, the annealed MoNi_4_/MoO_3−*x*_ exhibits three new peaks at 121, 480, and 719 cm^−1^ compared to the NiMoO_4_ precursor, and the three peaks could be ascribed to the lattice deformation mode, deformation of Mo=O stretching mode, and modification of the Mo_2_–O bond for MoO_3−*x*_ species, respectively. The high-resolution Mo 3d XPS spectra (Fig. 3D) of MoNi_4_/MoO_3−*x*_ further demonstrate the existence of Mo^0^, Mo^4+^, Mo^5+^, and Mo^6+^, and the low-valence states of Mo (Mo^5+^ and Mo^4+^) imply the formation of MoO_3−*x*_ with oxygen vacancies. As dual active components, the MoNi_4_/MoO_3−*x*_ hybrid exhibits a remarkable HER activity with low overpotentials of 17 mV at 10 mA cm^−2^ and 114 mV at 500 mA cm^−2^ (Fig. 3E), comparable to the values of the Pt catalyst (13 and 59 mV). The turnover frequency (TOF) is also estimated, and at an overpotential of 100 mV, the TOF of MoNi_4_/MoO_3−*x*_ is 1.13 s^−1^, much higher than that of Ni foam (0.06 s^−1^). After the 20 h chronoamperometry test at 20 or 30 mA cm^−2^, the decline of overpotential is negligible and the structure of nanorod arrays and composition remain almost unchanged, keeping the similar valence states of Mo^0^, Mo^4+^, and Mo^5+^. In order to investigate the origin of intrinsic activity, the Mo^0^ and Ni^0^ as well as Mo^4+^ and Mo^5+^ in MoNi_4_/MoO_3−*x*_ were oxidized to the high valence states of Mo^6+^ and Ni^2+^ by *in situ* electrochemical oxidation. As a result, HER activity decreases significantly (Fig. 3F), indicating that the activity of high valence molybdenum oxide is very low. Interestingly, the control sample annealed in an Ar atmosphere exhibits better activity than the completely oxidized sample due to the existence of little MoO_3−*x*_; however, since there is no MoNi_4_ alloy produced, its activity is still much lower than that of the samples obtained in a reducing atmosphere (Fig. 3G). Therefore, the MoNi_4_ alloy and MoO_3−*x*_ should synergistically catalyze the HER at the interface. Other MoNi_4_/MoO_3−*x*_ or MoNi_4_/NiMoO_*x*_ interfaces with different structures such as hollow, branch-leaf hierarchical, or nanowire structures have been prepared by reducing the NiMoO_4_ intermediate, and all exhibited excellent HER performance with the overpotentials from 29 to 38 mV at 10 mA cm^−2^, further demonstrating the unique advantages of Ni_4_Mo/MoO_*x*_ interfaces.^53–56^

**Fig. 4:**
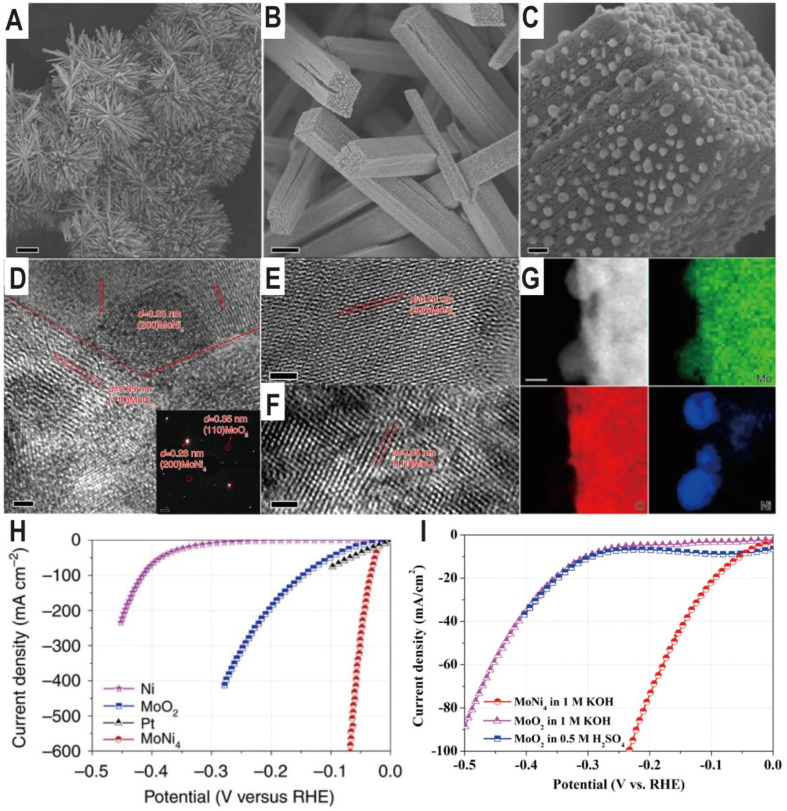
(A–C) Typical SEM images of MoNi_4_/MoO_2_@Ni. Scale bars, (A) 20 μm; (B) 1 μm; (C) 100 nm. (D–F) HRTEM images of MoNi_4_/MoO_2_@Ni. The inset image in (D) is the related selected-area electron diffraction pattern of the MoNi_4_ electrocatalyst and the MoO_2_ cuboids. Scale bars, (D–F) 2 nm; inset in (D), 1 1/nm. (G) Corresponding elemental mapping images of the MoNi_4_ electrocatalyst and the MoO_2_ cuboids. Scale bars: 20 nm. (H) Polarization curves of the MoNi_4_ electrocatalyst supported by the MoO_2_ cuboids, pure Ni nanosheets, and MoO_2_ cuboids on nickel foam. (I) Polarization curves of the MoO_2_ nanosheets and the MoNi_4_ electrocatalyst supported by the MoO_2_ cuboids on carbon cloth in different electrolytes. Reproduced with permission.^23^ Copyright 2017, Springer Nature.

The Ni_4_Mo/MoO_*x*_ interface structure of different components can be derived from the NiMoO_4_ intermediate by regulating the reduction temperature. The reduction temperature determines the reduction degree of Mo^6+^ ions in MoO_*x*_. For example, the Ni_4_Mo/MoO_2_ interface was obtained by reducing NiMoO_4_ cuboids in a H_2_/Ar atmosphere at 500 °C for 2 h.^23^ The NiMoO_4_ cuboids were grown beforehand on a piece of nickel foam *via* a hydrothermal reaction, and after reduction, MoNi_4_ nanoparticles were directly constructed on the surfaces of the MoO_2_ cuboids (Fig. 4A–C). The HRTEM images of the samples show lattice fringes with lattice distances of 0.35 and 0.28 nm, which correspond to the (110) facet of MoO_2_ and the (200) facet of MoNi_4_, respectively (Fig. 4D–F). The energy-dispersive X-ray spectroscopy (EDX) analysis indicates that the surface nanoparticles are constituted by only Mo and Ni with an atomic ratio of 1 : 3.84 (Fig. 4G), which well approaches 1 : 4. The polarization curves (Fig. 4H) show a zero onset potential and the overpotentials at current densities of 10 and 200 mA cm^−2^ for the Ni_4_Mo/MoO_2_ electrocatalyst were as low as 15 and 44 mV, respectively, which are significantly lower than the values for the Ni nanosheets, MoO_2_ cuboids, and commercial Pt/C. For the Pt/C electrocatalyst, it exhibits an overpotential of only 10 mV at a current density of 10 mV cm^−2^, but the maximum current density only reaches 80 mA cm^−2^ due to the Pt catalyst significantly peeling off from the support. In contrast, the three-dimensional (3D) cuboid array grown *in situ* on Ni foam enables the current density of Ni_4_Mo/MoO_2_ to reach 600 mA cm^−2^. Therefore, in addition to intrinsic activity, the 3D structure of the catalyst is also critical to the activity. Based on the electrochemical double-layer capacitances, the TOF of MoNi_4_/MoO_2_ is calculated to be 0.4 s^−1^ at the overpotential of 50 mV, which is higher than the TOF values of the previously reported Pt-free electrocatalysts. In addition, it can be seen from the polarization curve that MoO_2_ cuboids have a certain HER activity, which is much higher than that of Ni nanosheets. Interestingly, the HER activity of MoO_2_ loaded on carbon paper in acidic and alkaline media is very close (Fig. 4I), which indicates that the additional water dissociation in alkaline media does not reduce the reaction rate, and the overall low activity indicates that the binding capacity of H on MoO_2_ surface is poor. The Ni_4_Mo alloy just makes up for this shortcoming, and the combination of the two produces complementary and synergistic effects. The highly active Ni_4_Mo/MoO_2_ interface has also been demonstrated on other substrates such as stainless-steel wires obtained from worn-out tires (the overpotentials at the current densities of 100 and 200 mA cm^−2^ were only 63 and 77 mV, respectively).^57^

**Fig. 5:**
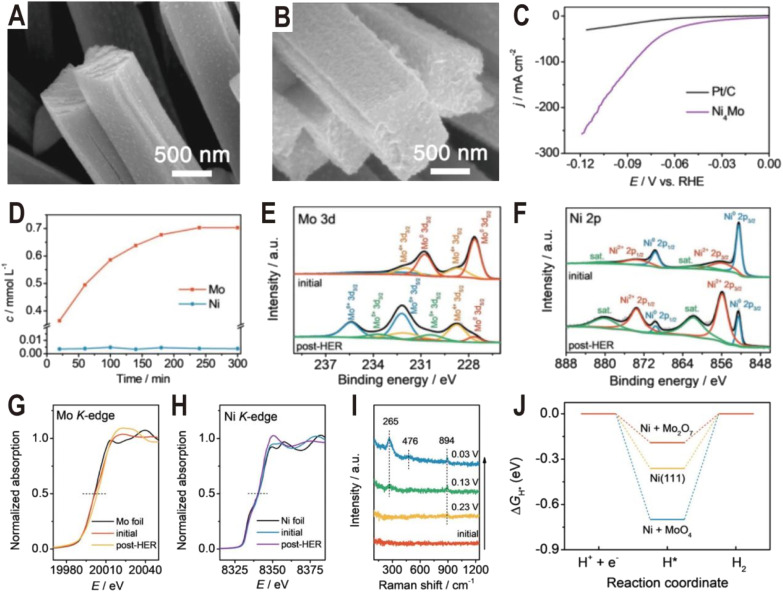
SEM images of the (A) as-prepared Ni_4_Mo nanorod arrays and (B) Ni_4_Mo nanorod arrays after the HER test for 12 h. (C) Polarization curves of the Ni_4_Mo alloy and Pt/C. (D) Time-dependent concentration of dissolved Mo and Ni in the electrolyte of the Ni_4_Mo alloy. (E) Mo 3d and (F) Ni 2p XPS spectra of Ni_4_Mo before and after the HER test. (G) Mo K-edge and (H) Ni K-edge XANES spectra of Ni_4_Mo before and after the HER test. (I) Potential-dependent *in situ* Raman spectra of Ni_4_Mo during the alkaline HER process. (J) Free energy diagrams of the HER on bare Ni(111), Ni(111) + MoO_4_, and Ni(111) + Mo_2_O_7_. Reproduced with permission.^58^ Copyright 2021, Wiley-VCH.

The Ni_4_Mo/MoO_*x*_ active interface can be generated not only by controlling the synthesis conditions but also in the electrochemical activation process. Typically, it is demonstrated that some Mo in Ni_4_Mo is oxidized during the activation process and dissolved in the form of MoO_4_^2−^.^58^ Then, the dissolved MoO_4_^2−^ will re-adsorb on the alloy surface and polymerize into the dimer Mo_2_O_7_^2−^ to form a highly active Ni_4_Mo (or Ni)/Mo_2_O_7_^2−^ interface. Fig. 5A and B show the SEM images of the as-prepared Ni_4_Mo nanorod arrays before and after the HER test. After the electrochemical activation, the nanorod array structure is nearly unchanged, except for the roughened surface and increased porosity. As shown in the polarization curves of Fig. 5C, the activated Ni_4_Mo displays an extremely low overpotential of only 86 mV at 100 mA cm^−2^, which is much better than that of commercial 20% Pt/C with the same mass loading. By analyzing the content of Mo and Ni in the electrolyte, it is found that the content of Ni keeps at a low level throughout the activation process while the content of Mo increases with time and reaches the summit after 4 h (Fig. 5D). The Mo 3d XPS spectra (Fig. 5E) show that the peaks of Mo^0^ obviously decrease after the HER test, while the peaks of Mo^4+^, Mo^5+^, and Mo^6+^ notably increase, indicating the oxidation of Mo. Similarly, in the Ni 2p XPS spectra (Fig. 5F), the ratio of Ni^2+^ also increases. X-ray absorption near edge structure (XANES) is further employed to investigate the change of valence state of Mo and Ni. In the Mo K-edge spectra (Fig. 5G), the post-HER absorption edge is obviously larger than that of the initial one, confirming the oxidation of Mo, while for the Ni K-edge spectra (Fig. 5H), both the initial and the post-HER samples show a similar absorption edge to Ni foil, indicating that Ni is mainly zero valent in the reaction process. *In situ* Raman spectra are then used to explore the change of Mo species during HER activation (Fig. 5I). Initially, there is no Raman signal, and after applying a voltage of 0.23 V, a peak centered at 894 cm^−1^ first appears, which can be assigned to Mo=O symmetric stretching vibration in the MoO_4_ tetrahedron, indicating the production of MoO_4_^2−^ ions. Interestingly, with the potential decreasing, another two peaks at 265 and 476 cm^−1^ arise and become strong, both of which correspond to the deformation mode and symmetric stretching mode of Mo–O–Mo, respectively. The appearance of these two new peaks indicates the possible polymerization of MoO_4_^2−^ into the dimer of Mo_2_O_7_^2−^. The DFT calculations further demonstrate that the Ni (Ni_4_Mo)/Mo_2_O_7_^2−^ interface optimizes the bonding energy with the adsorbed hydrogen (Fig. 5J).

## 3. Ni_*x*_Mo_*y*_ alloy/hydroxide interfaces

**Fig. 6:**
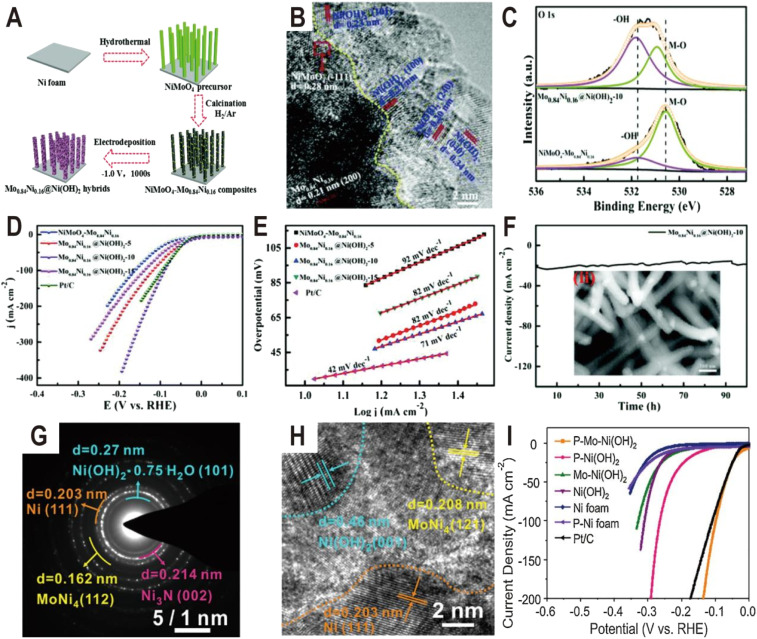
(A) Schematic illustration of the preparation of the Mo_0.84_Ni_0.16_@Ni(OH)_2_ heterostructure. (B) HRTEM image of Mo_0.84_Ni_0.16_@Ni(OH)_2_. (C) The high-resolution XPS spectra of O 1s for NiMoO_4_–Mo_0.84_Ni_0.16_ composites and the Mo_0.84_Ni_0.16_@Ni(OH)_2_ heterostructure. (D and E) Polarization curves and the corresponding Tafel plots of NiMoO_4_–Mo_0.84_Ni_0.16_, and Mo_0.84_Ni_0.16_@Ni(OH)_2_ with different deposition times of 5, 10, and 15 min, and Pt/C. (F) The chronoamperometric curve at an overpotential of 34 mV for Mo_0.84_Ni_0.16_@Ni(OH)_2_. The inset is the SEM image after the stability test. Reproduced with permission.^59^ Copyright 2020, Royal Society of Chemistry. (G and H) SAED pattern and HRTEM image of P–Mo–Ni(OH)_2_ NSAs. (I) Polarization curves of Ni foam, P–Ni foam, Ni(OH)_2_ NSAs, Mo–Ni(OH)_2_ NSAs, P–Ni(OH)_2_ NSAs, P–Mo–Ni(OH)_2_ NSAs, and Pt/C. Reproduced with permission.^60^ Copyright 2020, Elsevier.

Except oxides, metal hydroxides such as Ni(OH)_2_ and Co(OH)_2_ are another kind of active component to promote the dissociation of water for the HER. For example, the Ni(OH)_2_ modified Mo_0.84_Ni_0.16_ alloy exhibited significantly enhanced activity for the HER compared to the unmodified alloy.^59^ The synthesis process of Mo_0.84_Ni_0.16_@Ni(OH)_2_ hybrids is illustrated in Fig. 6A. First the NiMoO_4_ nanowire precursors were grown on Ni foam by a facile hydrothermal process and then were calcined in a H_2_/Ar atmosphere to obtain the NiMoO_4_–Mo_0.84_Ni_0.16_ composites. Subsequently, the Ni(OH)_2_ nanosheets were modified on the surface of the composites by electrodeposition. As a result, the Mo_0.84_Ni_0.16_ alloy and Ni(OH)_2_ nanosheets formed a heterostructure (Mo_0.84_Ni_0.16_@Ni(OH)_2_). The HRTEM image of the interface (Fig. 6B) demonstrates the lattice distance of 0.27, 0.23, 0.20, and 0.34 nm, which corresponds to the (100), (101), (240), and (040) planes of Ni(OH)_2_, respectively, and the crystalline lattice distance of 0.24 nm and 0.28 nm corresponds to the (111) plane of Mo_0.84_Ni_0.16_ and (−111) plane of NiMoO_4_, respectively. In the O 1s XPS spectra (Fig. 6C), the intensity of the OH^−^ peak becomes strong in Mo_0.84_Ni_0.16_@Ni(OH)_2_ compared to NiMoO_4_–Mo_0.84_Ni_0.16_ and both O 1s peaks also exhibit a positive shift, suggesting the strong electronic interaction between Mo_0.84_Ni_0.16_ and Ni(OH)_2_. As expected, due to the abundant interface structure and synergistic effect between Mo_0.84_Ni_0.16_ and Ni(OH)_2_, the Mo_0.84_Ni_0.16_@Ni(OH)_2_ heterostructure exhibited high HER activity with a very small overpotential at 10 mA cm^−2^ (10 mV), low Tafel slope (71 mV dec^−1^), and excellent stability (100 h) (Fig. 6D–F), which is much better than that of unmodified NiMoO_4_–Mo_0.84_Ni_0.16_. After the 100 h stability test, the SEM image of Mo_0.84_Ni_0.16_@Ni(OH)_2_ (the inset of Fig. 6F) displays the consistent surface morphology with the original Mo_0.84_Ni_0.16_@Ni(OH)_2._ Another more complex heterostructure is composed of Ni_4_Mo, Ni_3_N, Ni, and Ni(OH)_2_ (Fig. 6G and H), which was synthesized by low-temperature H_2_/N_2_ plasma activation of Mo-doped Ni(OH)_2_ nanosheets arrays (P–Mo–Ni(OH)_2_ NSAs).^60^ Due to the synergistic effect of different components, especially the interaction between Ni_4_Mo and Ni(OH)_2_, the optimized heterostructure only needs a low overpotential of 22 and 98 mV to deliver a current density of 10 and 100 mA cm^−2^ (Fig. 6I), respectively. At an overpotential of 50 mV, the TOF value of P–Mo–Ni(OH)_2_ is 1.325 s^−1^, which is much larger than that of Ni (OH)_2_ (0.42 s^−1^). After the 100 h stability test at an overpotential of 22 mV, the HRTEM image, XRD pattern, and XPS spectra imply that metallic Ni, Ni_4_Mo alloy, Ni_3_N, and Mo incorporated Ni(OH)_2_ still exist. Moreover, it is found that the Mo concentration in the electrolyte first increases from 0 to 1.13 ppm and then remains relatively stable, and except for the dissolution of a certain amount of Mo and N and the oxidation of some amount of metallic Ni, the chemical composition and valence states of the P–Mo–Ni(OH)_2_ change little during the HER. Theoretical and experimental studies show that the metallic Ni_4_Mo optimizes the Gibbs free energy for hydrogen adsorption and Ni(OH)_2_ can weaken the HO–H bond of absorbed water to facilitate water dissociation in the hetero-interface of Ni_4_Mo/Ni(OH)_2_.

**Fig. 7:**
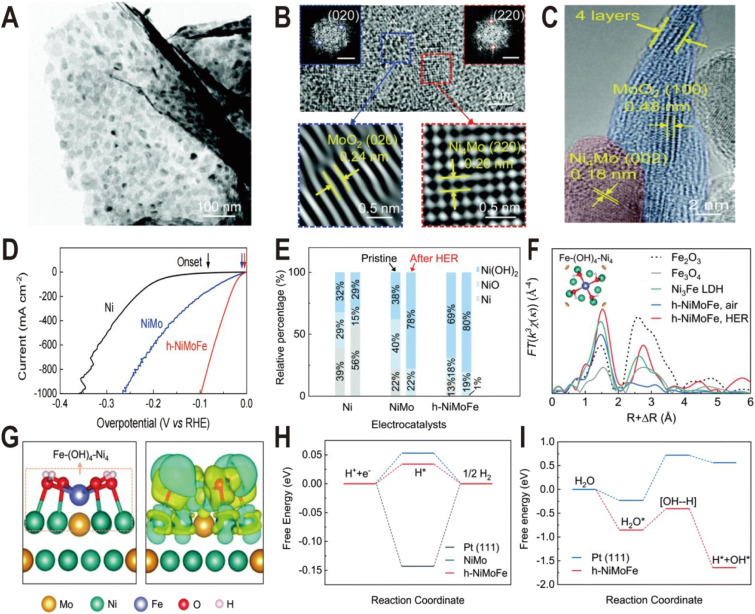
(A) TEM image of the h-NiMoFe catalyst. (B and C) HRTEM images of h-NiMoFe. (B) An enlarged view of h-NiMoFe, lattice fringes of a MoO_2_ nanosheet (blue box) and Ni_4_Mo nanoparticles (red box), scale bars in the insets are 5 1/nm; (C) side view of the MoO_2_ nanosheet. (D) Polarization curves of h-NiMoFe and its control samples. (E) Relative percentages of surface Ni species from the Ni 3s XPS spectra on the Ni, NiMo, and h-NiMoFe samples before and after the HER test. (F) FT-EXAFS of the h-NiMoFe catalyst and control samples at Fe K-edges. (G) Calculated relaxed configuration of an Fe–(OH)_4_–Ni_4_ motif on a Ni_4_Mo (002) slab and the corresponding charge density difference in this configuration. (H and I) Adsorption energies for H and dissociated H_2_O on h-NiMoFe and control samples. Reproduced with permission.^50^ Copyright 2021, Royal Society of Chemistry.

Ni(OH)_2_ on the NiMo alloy surface can not only be modified additionally but also be produced *in situ* by introducing Fe ions. It has been demonstrated that the presence of Fe on the surface of the Ni_4_Mo alloy can induce the conversion of the Ni to Ni(OH)_2_ by forming the Fe–(OH)_4_–Ni_4_ motif.^50^ This catalyst is composed of hydroxide-mediated Ni_4_Mo nanoparticles decorated with FeO_*x*_ and anchored onto MoO_2_ nanosheets (h-NiMoFe). It was prepared by a two-step method: Fe-doped NiMoO_4_ microsphere precursors (Fe–NiMoO_4_) were first grown on Ni foam by the hydrothermal method and then reduced at 500 °C in a Ar/H_2_ atmosphere. The TEM image (Fig. 7A) shows that the catalyst is composed of ultrathin nanosheets with nanoparticles anchored onto them and the HRTEM images (Fig. 7B and C) show typical lattice spacings of 0.24 nm and 0.20 nm, corresponding to the (020) plane of MoO_2_ and the (220) plane of Ni_4_Mo, respectively, and no Fe-based compounds are detected. The polarization curves in Fig. 7D show that the activity of h-NiMoFe is much better than that of NiMo and Ni samples, indicating that alloying of Ni_4_Mo and addition of Fe play vital roles in the good performance of the h-NiMoFe catalyst. The overpotential of h-NiMoFe at 10 mA cm^−2^ is only 14 mV and even at an increased current density of 1000 mA cm^−2^, the overpotential is still very low (98 mV). After 40 h continuous operation, h-NiMoFe shows good stability and maintains its crystalline structure well. In the Ni 3s XPS spectra, it is found that there is a higher content of surface hydroxide on h-NiMoFe than those on the control NiMo and Ni samples due to the introduction of Fe (Fig. 7E). *In situ* XAS characterization results indicate that a new chemical species containing the Fe–O(H)–Ni motif forms and FT-EXAFS fitting results show that an iron site is coordinated by four O atoms (or OH groups) and four Ni atoms. Density functional theory (DFT) calculations confirm the local structure of one Fe connecting to four hydroxylated Ni sites on the Ni_4_Mo surface (denoted as Fe–(OH)_4_–Ni_4_, Fig. 7F and G). The added iron in h-NiMoFe extracts electrons from Ni atoms to lead to more unoccupied states of Ni sites, which remarkably changes the charge distribution of Ni sites. The comparison of PDOS shows that the d-band centers of Ni in h-NiMoFe exhibit more negative energy than the NiMo catalyst, indicating a stronger H bonding behavior. The energy diagrams of Gibbs free energies further show nearly 0 eV adsorption free energy of H (Δ*G*_H_) and a much lower energy barrier and fast kinetics for water dissociation for h-NiMoFe compared to Pt (Fig. 7H and I). In addition to Ni(OH)_2_, this system also contains MoO_2_, which can also promote water dissociation as discussed above, so this system may be a catalytic system with synergistic effects of two interfaces: Ni_4_Mo/Ni(OH)_2_ and Ni_4_Mo/MoO_2_.

**Fig. 8:**
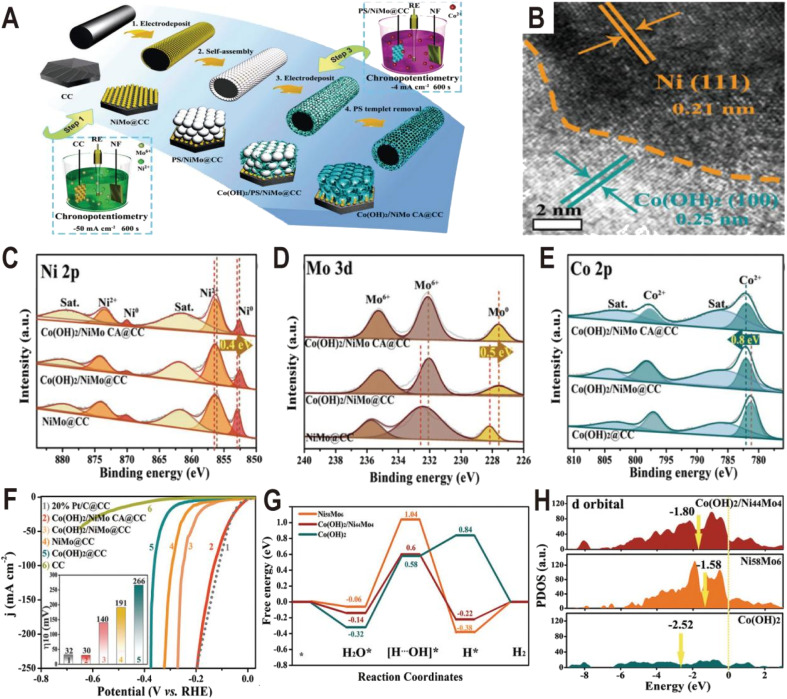
(A) Synthetic illustration of the fabrication process of Co(OH)_2_/NiMo CA@CC. (B) HRTEM image of NiMo alloy particles from Co(OH)_2_/NiMo CA@CC. (C–E) XPS spectra of Ni 2p, Mo 3d, and Co 2p of Co(OH)_2_/NiMo CA@CC, Co(OH)_2_/NiMo@CC, NiMo@CC, and Co(OH)_2_@CC. (F) Polarization curves of bare CC, Co(OH)_2_/NiMo CA@CC, Co(OH)_2_/NiMo@CC, NiMo@CC, Co(OH)_2_@CC, and Pt/C@CC toward the HER in 1.0 M KOH. (G) The free energy diagram for the HER on the surface of Ni_58_Mo_6_ and Co(OH)_2_, and the interface of Co(OH)_2_/Ni_44_Mo_4_. (H) The projected density-of-states of d orbitals of Ni_58_Mo_6_, Co(OH)_2_, and Co(OH)_2_/Ni_44_Mo_4_ with aligned Fermi levels. Reproduced with permission.^61^ Copyright 2021, Wiley-VCH.

Similar to Ni(OH)_2_, Co(OH)_2_ is also used to modify the NiMo alloy to improve the HER performance. The Co(OH)_2_ cavity array-encapsulated NiMo alloy on carbon cloth (Co(OH)_2_/NiMo CA@CC) was prepared by a two-step electrodeposition route.^61^ As shown in Fig. 8A, the NiMo alloy was first deposited on carbon cloth by chronopotentiometry (defined as NiMo@CC). And then, with the assistance of a layer of polystyrene (PS) template, Co(OH)_2_ was confined to nucleate and grow around the PS microspheres by the second electrodeposition. After dissolving the PS template, the uniform and ordered Co(OH)_2_ cavity array was obtained on the surface of NiMo@CC (defined as Co(OH)_2_/NiMo CA@CC). The NiMo alloy is composed of uniform nanoparticles with an average size of ∼70 nm and these nanoparticles are connected to the Co(OH)_2_ thin nanosheets. The HRTEM image at the boundary shows lattice fringes with a distance of 0.21 and 0.25 nm (Fig. 8B), corresponding to the (111) plane of Ni metal and the (100) plane of α-Co(OH)_2_, respectively, suggesting that metallic Ni as the dominant phase exists in the Ni_*x*_Mo_*y*_ alloy and forms a heterojunction interface with Co(OH)_2_. From the XPS spectra (Fig. 8C–E), it is found that both the Ni 2p peaks and Mo 3d peaks in Co(OH)_2_/NiMo CA@CC are shifted by 0.4 and 0.5 eV toward the lower binding energy relative to those of NiMo@CC, respectively, while the characteristic peaks of Co 2p are shifted toward high binding energy by 0.8 eV (Fig. 8E), indicating the transfer of electrons from Co of Co(OH)_2_ to the surface of the NiMo alloy. The HER polarization curves (Fig. 8F) show that Co(OH)_2_/NiMo CA@CC exhibits the highest catalytic activity among NiMo@CC, Co(OH)_2_@CC, and NiMo/Co(OH)_2_@CC with an overpotential of 30 mV to reach 10 mA cm^−2^. The comparative experiments demonstrate that the coupling of NiMo with Co(OH)_2_ and the cavity array structure can effectively boost the HER activity. The Gibbs free energy diagram (Fig. 8G) shows that water is more easily adsorbed on Co(OH)_2_ than on NiMo alloy, and the energy barrier to break the H–OH bond is only 0.58 eV on Co(OH)_2_, which is much lower than that of the NiMo alloy (up to 1.04 eV), indicating that the introduction of Co(OH)_2_ substantially accelerates the kinetics of the water adsorption and dissociation steps. Moreover, from the PDOS of d orbitals (Fig. 8H), the d-band center of Co(OH)_2_/NiMo (−1.80 eV) is downshifted relative to the single NiMo (−1.58 eV), which can weaken the bonding strength of H and thus promote the H_2_ evolution.

**Fig. 9:**
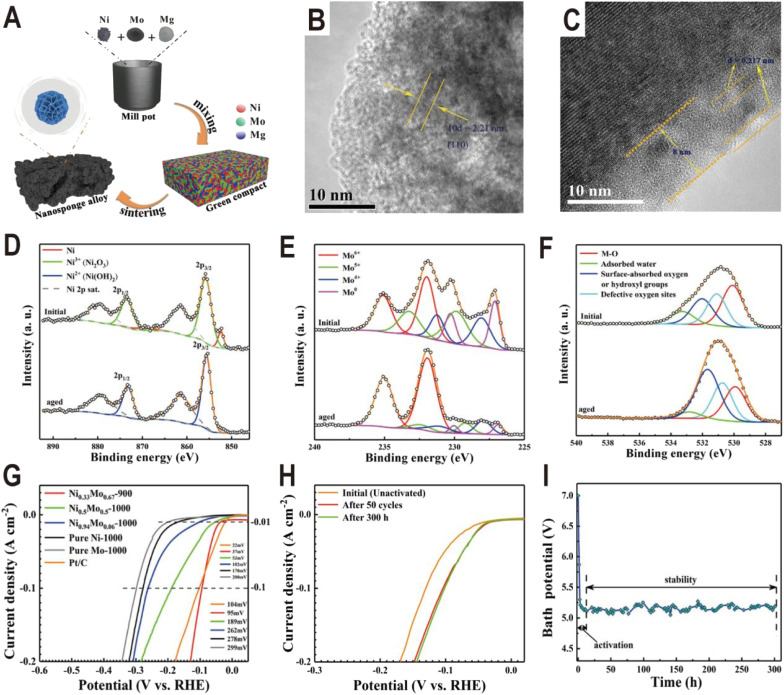
(A) Schematic diagram of the fabrication process for a nanosponge-like NiMo solid solution prepared by high-temperature sintering. (B and C) HRTEM images of Ni_0.33_Mo_0.67_-900 before and after the HER test. (D–F) Ni 2p, Mo 3d, and O 1s XPS spectra of Ni_0.33_Mo_0.67_-900 before and after aging. (G) Polarization curves of pure Ni-1000, pure Mo-1000, Ni_0.94_Mo_0.06_-1000, Ni_0.5_Mo_0.5_-1000, Ni_0.33_Mo_0.67_-900, and Pt/C electrodes in a 1 M KOH solution. (H) Comparison of polarization curves of Ni_0.33_Mo_0.67_-900 obtained under different conditions. (I) Bath voltage variations at a current density of 2 A cm^−2^ in 1 M KOH solution. Reproduced with permission.^62^ Copyright 2020, American Chemical Society.

In addition to the interfaces of controllable synthesis, the Ni_*x*_Mo_*y*_ alloy/hydroxide interface can also be generated during electrochemical activation. For the Ni_*x*_Mo_*y*_ alloys obtained by high-temperature metallurgy, their HER activities are generally not high due to the lack of an interface structure, but their activities will be greatly improved after electrochemical activation to produce a layer of Ni(OH)_2_ or MoO_*x*_ on the surface. A typical example is the nanosponge-like Ni_0.33_Mo_0.67_ solid solution catalyst synthesized *via* a one-step high-temperature (900 °C) sintering method by using metallic Ni, Mo, and magnesium (Mg) powders (Fig. 9A).^62^ Spherical Mg powder was added as a pore-making agent to prepare nanoporous alloys. The HRTEM image of the Ni_0.33_Mo_0.67_-900 solid solution shows an interplanar spacing of 0.221 nm (Fig. 9B), corresponding to the (110) facet of the metallic Mo body-centered cubic (bcc) structure. Interestingly, after an activation of 200 cycles with a scan rate of 10 mV s^−1^ and an overpotential range of 0–0.447 V (*vs.* RHE), an (oxy)hydroxide layer with a thickness of approximately 8 nm was clearly formed on the surface of Ni_0.33_Mo_0.67_-900, as shown in Fig. 9C. XPS spectra further prove the change of surface composition before and after activation. For Ni 2p (Fig. 9D), after aging, the peaks of Ni^0^ and Ni^3+^ at 852.4 eV and 855.8 eV disappear and the peak of Ni^2+^ at 855.6 eV is produced, corresponding to a phase of Ni(OH)_2_. For Mo 3d (Fig. 9E), the relative amount of Mo^0^, Mo^4+^, and Mo^5+^ species in the activated sample rapidly decreases relative to the pristine sample; in contrast, Mo^6+^ increases, indicating the formation of high valence MoO_*x*_. For O 1s (Fig. 9F), the peak intensity of hydroxyl groups has an obvious increase. The polarization curves in Fig. 9G show that the activated Ni_0.33_Mo_0.67_-900 solid solution catalyst exhibits a lower overpotential of 37 mV at a current density of 10 mA cm^−2^, and the performance of the sample without activation is obviously lower than that of the sample (Fig. 9H). The stability test curve also shows an obvious activation process, and the bath voltage demonstrates a fast decrease in the initial period of electrolysis, as shown in Fig. 9I. After activation, the Ni_0.33_Mo_0.67_-900 solid solution possesses an ultrahigh stability (>300 h) at 2 A cm^−2^ in a 1 M KOH electrolyte. As another example, the plasma sprayed RANEY®-type NiMo electrode also forms a layer of Ni(OH)_2_ and MoO_*x*_ after chemical activation.^63^ Due to proper micropore/macropore distribution, appropriate amounts of active nickel and molybdenum species, and the oxidation degree, the activated NiMo electrode delivered a high current density of 200 mA cm^−2^ at 82 mV and stabilized with no measurable degradation over 47 days for the HER in 30 wt% KOH.

From the above example analysis, it can be seen that the Ni_*x*_Mo_*y*_ alloy/hydroxide interface exhibits a bi-functional effect to simultaneously promote the dissociation of water and the combination of adsorbed hydrogen. The kinetics of the HER in alkaline solutions will depend both on the rate of desorption of OH_ad_ to accommodate the adsorption of H_2_O on hydroxide and on the rate of H_ad_ recombination, which is optimized on the alloy. For the dissociation of water, the activity will be controlled by the bond strength between metal hydroxides M(OH)_*x*_ and OH_ad_. In order to explore their interaction, a descriptor, OH–M^2+*δ*^ bond strength (0 ≤ *δ* ≤ 1.5), was proposed, and it was found that the activity trend was Mn < Fe < Co < Ni, which was inversely proportional to the OH–M^2+*δ*^ strength of Ni < Co < Fe < Mn.^64^ For the Fe and Mn hydroxides, due to their strong bonding with OH_ad_, OH_ad_ cannot be desorbed and the reaction cannot continue, resulting in ‘poisoning’ of the surface. In contrast, for Ni hydroxides that bind OH_ad_ neither too strongly nor too weakly, the maximum activity was realized for the HER. Thus, the overall rate of the HER may, in principle, be controlled by optimizing the density and the nature of the sites required for dissociation of water on M(OH)_*x*_, as well as the OH–M^2+*δ*^ and alloy-H_ad_ energetics.

## 4. Conclusions and perspectives

In this perspective, we systematically summarize the characteristics of structures and components obtained by different synthesis methods in Ni–Mo-based catalysts, and discuss their corresponding relationship with HER performance in detail. It can be found that most of the currently reported highly active Ni–Mo-based catalysts have alloy–oxide or alloy–hydroxide interface structures, as summarized in Tables 1 and 2. According to the logic of synthesis–structure–performance, we discuss the influence of the composition changes of two types of interface structures on HER activity. For the alloy–oxide interfaces, the Ni_4_Mo/MoO_*x*_ (mixed oxide with the Mo ion valence from +4 to +6) composites produced by electrodeposition or hydrothermal combined with thermal reduction exhibit activities close to that of platinum. For only the alloy or oxide, their activities are significantly lower than that of composite structures, indicating the synergistic catalytic effect of binary components. Interestingly, the HER activity of MoO_2_ in acidic and alkaline media is very close, indicating that MoO_2_ can eliminate the influence of additional water dissociation under alkaline conditions. For the alloy–hydroxide interfaces, the activity of the Ni_*x*_Mo_*y*_ alloy with different Ni/Mo ratios is greatly improved by constructing heterostructures with hydroxides such as Ni(OH)_2_ or Co(OH)_2_. In particular, pure alloys obtained by metallurgy must be activated to produce a layer of mixed Ni(OH)_2_ and MoO_*x*_ on the surface to achieve high activity. Theoretical and experimental studies show that metallic Ni_*x*_Mo_*y*_ alloys optimize the Gibbs free energy for hydrogen adsorption and MoO_*x*_ or Ni(OH)_2_ can weaken the HO–H bond of absorbed water to facilitate water dissociation in the hetero-interfaces. Therefore, it can be seen that the activity of Ni–Mo catalysts probably originates from the interfaces of alloy–oxide or alloy–hydroxide.

**Table 1:** Electrochemical performances of highly active Ni–Mo-based catalysts with alloy–oxide interfaces in 1 M KOH

Electrocatalyst	Synthesis method	Composition and structure	Onset potential (mV)	Overpotential (mV)	TOF (s^−1^)	Tafel slope (mV per decade)	Stability	Ref.
Ni_4_Mo/MoO_*x*_/Cu	Electrodeposition	Ni_4_Mo, MoO_*x*_, nanosheets	0	*η*_10_ = 16, *η*_250_ = 105 (70 °C, 30% KOH)	—	64	10 mA cm^−2^, 24 h, 50 mA cm^−2^, 24 h, 100 mA cm^−2^, 24 h	51
MoNi_4_/MoO_3−*x*_	Hydrothermal and reduction @ 350 °C	MoNi_4_, MoO_3−*x*_, NiMoO_4_, nanorods	5	*η*_10_ = 17, *η*_100_ = 52, *η*_500_ = 114	1.13 @ *η*_100_	36	20 and 30 mA cm^−2^ for total 20 h	52
MoNi_4_/MoO_2_@NF	Hydrothermal and reduction @ 500 °C	MoNi_4_, MoO_2_, cuboids	0	*η*_10_ = 15, *η*_200_ = 44	0.4 @ *η*_50_	30	2000 CV cycles; 10, 100, and 200 mA cm^−2^ for total 30 h	23
MoNi_4_/SSW	Precipitation and decomposition	MoO_2_, MoNi_4_	—	*η*_100_ = 63, *η*_200_ = 77, *η*_500_ = 115 (5 M KOH, 353 K), *η*_1000_ = 161 (5 M KOH, 353 K)	—	40	100 mA cm^−2^, 18 h, 150 mA cm^−2^, 150 h (5 M KOH, 353 K)	57
MoNi-HS	Hydrothermal and reduction @ 500 °C	Ni_4_Mo, MoO_*x*_, nanosheets	—	*η*_10_ = 38	—	31.4	10 mA cm^−2^, 10 h	53
NiMo/MoO_2_	Hydrothermal and reduction @ 450 °C	MoO_2_, NiMo, nanosheets	12	*η*_10_ = 52	1.06 @ *η*_150_	43.6	100 mA cm^−2^, 30 h	56
NC/NiMo/NiMoO_*x*_/NF	Hydrothermal and reduction @ 400 °C	Ni_4_Mo, MoO_3−*x*_, NiMoO_4_, nanowires	5	*η*_10_ = 29, *η*_200_ = 160	—	46	179 mA cm^−2^, 50 h	54
MoNi_4_/MoO_3−*x*_/NiCo@NF	Hydrothermal and reduction @ 350 °C	MoNi_4_, MoO_3−*x*_, NiCo, nanowires	—	*η*_10_ = 33, *η*_100_ = 149	—	34	3000 CV cycles; 20 mA cm^−2^, 24 h	55
Ni–Mo–O/Ni_4_Mo@NC	Electrodeposition, calcination, and electrodeposition	Ni_4_Mo, nanosheets	—	*η*_10_ = 61	—	99	10 mA cm^−2^, 15 h	24
NiMoFe@MoO_2_	Hydrothermal and reduction @ 500 °C	NiMoFe, MoO_2_, nano-pillars	—	*η*_10_ = 24, *η*_100_ = 63, *η*_200_ = 83, *η*_500_ = 130	—	33	10, 100, 200, 100, and 10 mA cm^−2^ for total 140 h	25
MoNi_4_@MoO_3−*x*_	Hydrothermal and reduction @ 550 °C	MoNi_4_, MoO_3−*x*_, nanorods	5.4	*η*_10_ = 58.6	—	44.8	10 mA cm^−2^, 10 h	37
NiMo M/O	Hydrothermal and reduction @ 500 °C	MoNi_4_, MoO_2_, nanosheets	—	*η*_10_ = 16, *η*_50_ = 50	—	31.9	10 mA cm^−2^, 11 h	28

**Table 2:** Electrochemical performances of highly active Ni–Mo-based catalysts with alloy–hydroxide interfaces in 1 M KOH

Electrocatalyst	Synthesis method	Composition and structure	Overpotential (mV)	TOF (s^−1^)	Tafel slope (mV per decade)	Stability	Ref.
Mo_0.84_Ni_0.16_@Ni(OH)_2_	Hydrothermal, reduction @ 500 °C and electrodeposition	NiMoO_4_, Mo_0.84_Ni_0.16_, Ni(OH)_2_, nanowires	*η*_10_ = 10, *η*_100_ = 91	0.93 @ *η*_100_	71	3000 CV cycles; 20 mA cm^−2^, 100 h	59
P–Mo–Ni(OH)_2_	Hydrothermal and low-temperature H_2_/N_2_ plasma activation	MoNi_4_, Ni(OH)_2_, Ni_3_N, MoNiN, nanosheet	*η*_10_ = 22, *η*_100_ = 98	1.325 @ *η*_50_	80	10 mA cm^−2^, 100 h, 50 mA cm^−2^, 50 h	60
Co(OH)_2_/NiMo CA@CC	Electrodeposition	Ni_*x*_Mo_*y*_, Co(OH)_2_, nanosheet	*η*_10_ = 30	—	41	10 mA cm^−2^, 24 h, 50 mA cm^−2^, 24 h, 100 mA cm^−2^, 24 h	61
h-NiMoFe	Hydrothermal and reduction @ 500 °C	MoO_2_, Ni_4_Mo, Fe–(OH)_4_–Ni_4_, nanosheet	*η*_10_ = 14, *η*_500_ = 74, *η*_1000_ = 97	2 @ *η*_50_	30.6	200, 600, 1000, and 1500 mA cm^−2^ for total 40 h	50
Ni_0.33_Mo_0.67_-900	High-temperature sintering	Ni_0.33_Mo_0.67_, MoO_*x*_, Ni(OH)_2_, nanosponge	*η*_10_ = 37, *η*_1000_ = 316	—	39.2	2 A cm^−2^, 300 h	62
RANEY®-type NiMo	Atmospheric plasma spraying and activation	MoNi, Mo_1.08_Ni_2.93_, Ni(OH)_2_, nanosponge	*η*_200_ = 82 (30 wt% KOH)	—	36	2 A, 47 days (30 wt% KOH)	63
NiMo/Ni(OH)_2_/CC	Hydrothermal and electrodeposition	NiMo, Ni(OH)_2_, nanosheet	*η*_10_ = 132	—	134.1	10 mA cm^−2^, 24 h	41
NiMo@Ni(OH)_2_MoO_*x*_	Electrodeposition	Ni(OH)_2_, MoO_3_, MoO_2_, nanoparticles	*η*_100_ = 160	—	115	10 mA cm^−2^, 24 h, 100 mA cm^−2^, 24 h	42

Although NiMo-based catalysts exhibit outstanding HER activity, the dissolvable nature of Mo in alkaline solution results in the poor stability of the Ni_*x*_Mo_*y*_ alloy due to the lower oxidation potential of Mo than that of H_2_. Therefore, controlling the dissolution of Mo species is crucial to improve the stability of NiMo-based catalysts. At present, there are three strategies that have proved effective and can be tried to develop: (1) preparation of the pure phase NiMo alloy with high specific surface area by a metallurgical method; the most stable catalysts reported are almost all treated by high-temperature alloying. Compared with nanoalloys prepared by a low-temperature chemical method, high-temperature alloying is beneficial to improve the compactness and continuity of the catalyst. After electrochemical activation, a layer of Ni(OH)_2_ or MoO_*x*_ on the surface is formed, which can not only facilitate the dissociation of water but also provide a protective layer to prevent the dissolution of the Mo element. (2) The formed interface structure is coated with carbon or other conductive oxides or hydroxides; by adsorbing or depositing a layer of carbon precursor molecules on the surface and then carbonizing, an alloy/oxide or alloy/hydroxide interface structure uniformly coated with carbon layer can be obtained. An additional protective layer will partially inhibit the dissolution of Mo. (3) Establishment of dynamic dissolution and re-adsorption equilibrium of Mo ions in electrolytes; the literature results reveal that MoO_4_^2−^ can be easily dissolved in KOH electrolyte and re-adsorbed on the surface of the catalyst during the oxygen evolution reaction (OER), which delivers a promoting effect on OER performance.^65^ Theoretical calculations show that the adsorption of the dimer Mo_2_O_7_^2−^ can promote the HER activity of metallic Ni.^58^ Therefore, the dissolution of Mo will be effectively inhibited and the stability of NiMo-based catalysts will be greatly improved through reasonable control of the above three aspects.

## Data availability

All data in this perspective were cited from other references.

## Author contributions

Z.-L. W. and Y. Y. conceived the topic and structure of the paper. All authors reviewed and contributed to this paper.

## Conflicts of interest

There are no conflicts to declare.

## Supplementary Material
